# Prevalence and correlates of irritability among U.S. adults

**DOI:** 10.1038/s41386-024-01959-3

**Published:** 2024-08-24

**Authors:** Roy H. Perlis, Ata Uslu, Jonathan Schulman, Aliayah Himelfarb, Faith M. Gunning, Nili Solomonov, Mauricio Santillana, Matthew A. Baum, James N. Druckman, Katherine Ognyanova, David Lazer

**Affiliations:** 1 Center for Quantitative Health, Massachusetts General Hospital, Boston, MA USA; 2 Department of Psychiatry, Harvard Medical School, Boston, MA USA; 3 Network Science Institute, Northeastern University, Boston, MA USA; 4 Institute for Quantitative Social Science, Harvard University, Boston, MA USA; 5 Department of Political Science, Northwestern University, Chicago, IL USA; 6 Department of Psychiatry, Weill Cornell Medicine, New York, NY USA; 7 John F. Kennedy School of Government and Department of Government, Harvard University, Cambridge, MA USA; 8 Department of Political Science, University of Rochester, Rochester, NY USA; 9 Department of Communication, School of Communication and Information, Rutgers University, New Brunswick, NJ USA

**Keywords:** Signs and symptoms, Psychology

## Abstract

This study aimed to characterize the prevalence of irritability among U.S. adults, and the extent to which it co-occurs with major depressive and anxious symptoms. A non-probability internet survey of individuals 18 and older in 50 U.S. states and the District of Columbia was conducted between November 2, 2023, and January 8, 2024. Regression models with survey weighting were used to examine associations between the Brief Irritability Test (BITe5) and sociodemographic and clinical features. The survey cohort included 42,739 individuals, mean age 46.0 (SD 17.0) years; 25,001 (58.5%) identified as women, 17,281 (40.4%) as men, and 457 (1.1%) as nonbinary. A total of 1218(2.8%) identified as Asian American, 5971 (14.0%) as Black, 5348 (12.5%) as Hispanic, 1775 (4.2%) as another race, and 28,427 (66.5%) as white. Mean irritability score was 13.6 (SD 5.6) on a scale from 5 to 30. In linear regression models, irritability was greater among respondents who were female, younger, had lower levels of education, and lower household income. Greater irritability was associated with likelihood of thoughts of suicide in logistic regression models adjusted for sociodemographic features (OR 1.23, 95% CI 1.22–1.24). Among 1979 individuals without thoughts of suicide on the initial survey assessed for such thoughts on a subsequent survey, greater irritability was also associated with greater likelihood of thoughts of suicide being present (adjusted OR 1.17, 95% CI 1.12–1.23). The prevalence of irritability and its association with thoughts of suicide suggests the need to better understand its implications among adults outside of acute mood episodes.

## Introduction

Most symptoms reflecting negative affective states, particularly those of depression and anxiety, are linked to one or a few diagnostic categories among adults in DSM-5. Irritability is an exception: while extreme irritability may reflect a manic episode, the symptom itself may be observed in almost any other psychiatric disorder [1]—and in individuals with no identified disorder at all [2].

When irritability is studied in modern psychiatry, it has often been as a feature of a psychiatric illness. Though it is not a core diagnostic symptom of major depression, around half of individuals with this diagnosis experience significant irritability [3], and twin studies indicate substantial a genetic correlation between depression and irritability [4, 5]. Such co-occurrence correlates with comorbid anxiety [3, 6, 7], poorer functioning and quality of life [6, 8], poorer treatment outcomes [9] and higher risk of suicidality [3, 10]. Whether irritability represents a feature of bipolar disorder in adults has led to disagreement in the literature: some studies suggested irritability could be best understood as a mixed episode feature [11], while another found no indication that these features are strongly associated with bipolarity per se [3].

Among children, irritability has also been investigated in less select samples [8] (reviewed in Leibenluft et al. [12]), with severe irritability observed in up to 5% [13]. The presence of irritability in children and adolescents has been shown to predict subsequent mood and anxiety diagnoses in 20-year follow-ups [14]. Corresponding studies in adults are lacking, however. The one prior epidemiologic study of irritability among adults asked a single question about irritability in the context of a past or current mood episode [7], precluding any population estimate. The NIMH Research Domain Criteria (RDoC) framework [15, 16] notes the importance of understanding how symptom dimensions vary across a continuum from health to disease; thus, a key gap in the literature remains how and where irritability manifests among adults in the general population. To address this gap, we drew on data from a large national survey of adults in the U.S., which asked questions about irritability, depression, and anxiety. We aimed first to characterize the prevalence of irritability in a nationally-representative, population-based cohort, and its sociodemographic correlates. As most prior work in adults focused on the co-occurrence of irritability and depression, we also investigated irritability specifically among individuals not meeting criteria for a probable major depressive episode. Next, we aimed to understand more broadly the relationship between irritability and other negative valence symptoms, investigating individual symptoms of depression and anxiety, and focusing in particular on thoughts of death and suicide. Finally, we investigated the prevalence and sociodemographic correlates of severe irritability specifically among adults not in the midst of a major depressive episode or probable generalized anxiety disorder. In aggregate, with these aims, we sought to better inform future clinical and research efforts to address irritability among adults.

## Methods

### Study design

We analyzed data drawn from two waves of the COVID States Project, a nonprobability [17] web-based survey conducted by a consortium of academic sites (COVIDstates.org) between November 2, 2023, and January 8, 2024. Primary analyses combined Wave 29, conducted between November 2 and December 2, 2023, and Wave 30, conducted between December 21, 2023, and January 26, 2024. Participants were individuals 18 and older residing in the United States who elected to take a general opinion survey through a commercial survey panel aggregator (PureSpectrum); they were drawn from all 50 U.S. states and the District of Columbia. To maximize representativeness, the survey applied state-level Census-based quotas intended to balance the sample on age, gender, race, and ethnicity. Participants provided written online consent before answering survey questions. The survey protocol was determined to be exempt by the Harvard University Institutional Review Board. We present results in accordance with AAPOR guidelines [18].

### Measures

#### Irritability

The Brief Irritability Test (BITe5) includes 5 questions beginning with, “Please indicate how often you have felt or behaved in the following ways, during the past two weeks, including today.” [19] For each item, frequency is reported on a 1–6 scale, from never to always, and item scores are summed to yield a total score between 5 and 30. Items were derived from a larger set to minimize overlap with depression, anger, and other related constructs; in a validation study it further demonstrated minimal effects of gender and strong internal consistency [19], and it has been employed in other survey-based investigations of negative affect (see, e.g., Li [20]). A recent psychometric review of irritability measures in young adults concluded that the BITe5 was optimal for this application [21].

#### Mood, anxiety, and thoughts of death and suicide

Depressive symptoms were assessed with the 9-item Patient Health Questionnaire (PHQ-9) to screen for depressive symptom severity [22, 23]. The 9 items reflect the individual diagnostic criteria for major depressive disorder in the DSM-5; respondents rate each symptom in terms of frequency over the prior 2 weeks on a 0–3 Likert-type scale (0 = not at all, 3 = nearly every day). A score of 10 or greater, representing at least moderate depression, was used to define a probable major depressive episode [22, 23]. Item 9 of this scale specifically assesses thoughts of death or suicide, asking about frequency of “[t]houghts that you would be better off dead, or of hurting yourself.” [24] Anxiety was measured using the 2-item Generalized Anxiety Disorder screen (GAD-2), derived from the 7-item version [25], which has been shown to have similar performance as a screening measure; a 3 or greater on this scale represents probable generalized anxiety.

#### Sociodemographic variables

We collected sociodemographic features by self-report. We asked survey respondents to identify race and ethnicity from a list including African American or Black, Asian American, Hispanic, Native American, Pacific Islander, white, or other, with the opportunity to provide a free text self-description. These categories were collected in order to confirm representativeness of the U.S. population, and are reported as advised in a recent guidance statement [26]. As in prior work, to facilitate the inclusion of smaller groups, we collapsed Native American, Asian-Pacific Islander, and other into a single category for analysis. Employment status was dichotomized to “working full-time” (yes vs. all others) as a dichotomous variable for consistency with prior work using this survey. Household socioeconomic status was collected by asking about annual household income, categorized as <$25k, $25–<$50k, $50k–<$100k, or $100k+ per year. Educational status was collected by asking respondents to select from highest level of formal education from among some high school or less, high school graduate, some college, undergraduate degree, or graduate degree.

### Statistical analysis

All analyses used R 4.3.2 [27]. For continuous measures we report mean and SD; for categorical variables, we report proportions and confidence intervals. In all regression models, we considered the following sociodemographic features: age in years, gender, education, annual household income, employment status, race and ethnicity, and rural, suburban, or urban setting based upon zip code. Extended regression models also included total depression severity as measured by PHQ-9 (or PHQ-8, where suicidal ideation was the dependent variable) and total anxiety severity as measured by GAD-2. As advised for nonprobability samples [28], we applied interlocking poststratification survey weights to approximate national demographic distributions (race and ethnicity, age, gender, educational level, region, and living in urban, suburban, or rural areas) as determined by 2020 US Census American Community Survey results [29], using the R survey package [30] (version 4.2–1). For participants who responded to more than one survey wave, the initial (index) survey was included; analyses of prior waves adopting alternate approaches (random selection of a wave, or including all observations as nested within an individual) yielded similar results [31]. In light of very low rates of missing data (Table 1), we did not apply multiple imputations. For all analyses, two-tailed *p* < 0.05 was considered to represent statistical significance.

**Table 1:** Characteristics of survey respondents with or without moderate or greater depressive symptoms.

	Less than moderate (*N* = 30,588)	Moderate or greater (*N* = 12,151)	Total (*N* = 42,739)	*p*-value
Age (years)	48.9 (17.1)	38.6 (14.2)	46.0 (17.0)	<0.001
Gender				<0.001
Female	17,456 (57.1%)	7545 (62.1%)	25,001 (58.5%)	
Male	12,934 (42.3%)	4347 (35.8%)	17,281 (40.4%)	
Nonbinary	198 (0.6%)	259 (2.1%)	457 (1.1%)	
Race and Ethnicity				<0.001
African American	4180 (13.7%)	1791 (14.7%)	5971 (14.0%)	
Asian American	850 (2.8%)	368 (3.0%)	1218 (2.8%)	
Hispanic	3565 (11.7%)	1783 (14.7%)	5348 (12.5%)	
Native American	424 (1.4%)	203 (1.7%)	627 (1.5%)	
Other Race	434 (1.4%)	197 (1.6%)	631 (1.5%)	
Pacific Islander	335 (1.1%)	182 (1.5%)	517 (1.2%)	
White	20,800 (68.0%)	7627 (62.8%)	28,427 (66.5%)	
Education				<0.001
Some high school or less	914 (3.0%)	739 (6.1%)	1653 (3.9%)	
High school graduate	6764 (22.1%)	3638 (29.9%)	10,402 (24.3%)	
Some college	7646 (25.0%)	3558 (29.3%)	11,204 (26.2%)	
College degree	11,157 (36.5%)	3374 (27.8%)	14,531 (34.0%)	
Graduate degree	4107 (13.4%)	842 (6.9%)	4949 (11.6%)	
Employment				<0.001
Full-time	12,139 (39.7%)	4698 (38.7%)	16,837 (39.4%)	
Household income				<0.001
<$25k	5724 (18.7%)	3801 (31.3%)	9525 (22.3%)	
$25–<$50k	8133 (26.6%)	3550 (29.2%)	11,683 (27.3%)	
$50–<$100k	10,470 (34.2%)	3371 (27.7%)	13,841 (32.4%)	
$100k+	6261 (20.5%)	1429 (11.8%)	7690 (18.0%)	
Urbanicity				<0.001
Rural	5173 (16.9%)	2394 (19.7%)	7567 (17.7%)	
Suburban	17,685 (57.8%)	6664 (54.8%)	24,349 (57.0%)	
Urban	7730 (25.3%)	3093 (25.5%)	10,823 (25.3%)	
PHQ9 total	3.4 (2.9)	15.8 (4.7)	6.9 (6.6)	<0.001
Thoughts of death/suicide	2039 (6.7%)	6916 (56.9%)	8955 (21.0%)	<0.001
GAD2 total	0.9 (1.2)	3.8 (1.7)	1.7 (1.9)	<0.001
BITe5 total	11.6 (4.1)	18.8 (5.5)	13.6 (5.6)	<0.001
Repeated survey	1879 (6.1%)	416 (3.4%)	2295 (5.4%)	<0.001

*PHQ9* patient health questionnaire, 9 item, *GAD2* generalized anxiety disorder, 2 item, *BITe5* brief irritability test, 5 item.

We first examined associations between irritability and individual sociodemographic features as specified above via univariate and multivariable linear regression models, to understand potential correlates of irritability. We adopted the same approach to examine associations between total irritability score and total depression and anxiety scores, again in univariate and multivariable (sociodemographic-adjusted) linear regression models.

Beyond total scores, we then examined simple correlations between individual irritability, depression, and anxiety items. As a means of understanding higher-order relationships among these items (i.e., beyond bivariate correlations), we applied network analysis, visualizing symptom networks in individuals with moderate or greater depressive symptoms, and in those with less than moderate symptoms (i.e., no depression or mild depression). Symptoms were visualized using the igraph package [32] as enhanced by Ognyanova [33]; networks were contrasted using permutation to determine if they were significantly different in structure.

We then investigated the nature of the relationship between suicidal ideation, measured by PHQ-9 item 9, and irritability. These analyses utilized linear regression, both univariate and multivariable with adjustment for sociodemographic features. While all other analyses treated survey data cross-sectionally (i.e., with one observation per participant), we then investigated whether irritability preceded emergence of suicidal ideation. To do so, we derived a sub-cohort from the total group, defined by individuals who responded to survey Wave 29 and returned for Wave 30 (*n* = 2295), and whose PHQ-9 item 9 score at Wave 29 was 0 (*n* = 1979; 86.2%), indicating no suicidal ideation or thoughts of death at initial survey. After characterizing this group descriptively, we used logistic regression to examine association irritability at initial survey and new onset of suicidal ideation at subsequent survey, adjusting for sociodemographic features.

Finally, we investigated the characteristics of individuals who experience high levels of irritability outside of a probable major depressive episode or positive screen for generalized anxiety, defined as individuals with PHQ-9 less than 10 (i.e., less than moderate depression) and GAD-2 less than 3 (i.e., not screen-positive for generalized anxiety disorder) and irritability in the top decile of symptoms across the population. These analyses again applied linear regression to investigate associations between sociodemographic features and irritability.

## Results

The cohort as a whole included 42,739 individuals, mean age 46.0 (SD 17.0) years; 25,001 (58.5%) identified as women, 17,281 (40.4%) as men, and 457 (1.1%) as nonbinary. A total of 1,218 (2.8%) identified as Asian, 5971 (14.0%) as Black, 5348 (12.5%) as Hispanic, 1775 (4.2%) as another race, and 28,427 (66.5%) as white. Mean irritability score on the BITe-5 was 13.6 (SD 5.6) on a scale from 5 to 30; Cronbach’s alpha for this scale was 0.92 (95% CI 0.92–0.92), indicating excellent internal consistency. Additional characteristics of the cohort are summarized in Table 1, divided by presence/absence of moderate or greater major depressive symptoms for comparability with prior studies that focused solely on irritability during major depressive episodes.

**Fig. 1:**
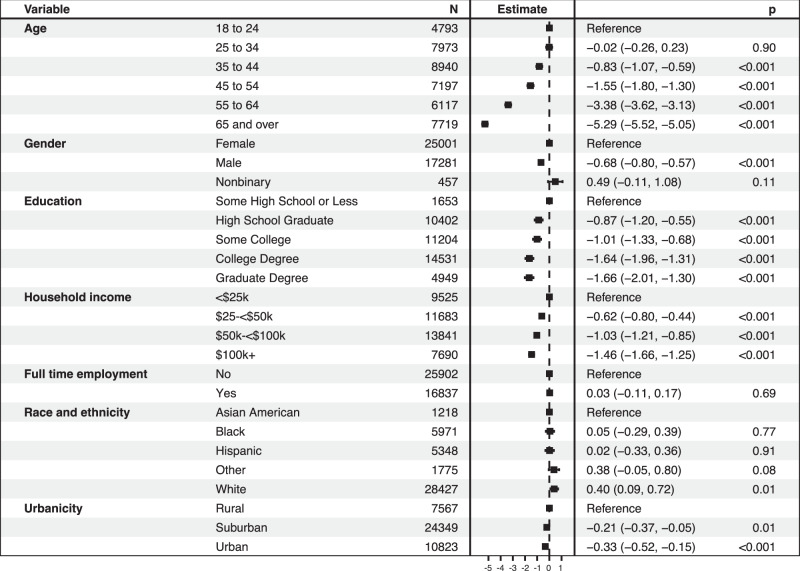
Linear regression model of irritability associated with sociodemographic and clinical features.

We first examined the sociodemographic features associated with irritability, in univariate (Table S1) and then multivariable linear regression models (Fig. 1). Overall, female respondents, those who were younger, those with lesser levels of education, those with lower household income, and those who were white reported greater levels of irritability in adjusted models. In additional regression models, total depression severity was associated with greater levels of irritability (unadjusted coefficient 0.59, 95% CI 0.59–0.60; adjusted for sociodemographic features 0.55 (95% CI 0.54–0.56), as was total anxiety severity (unadjusted coefficient 2.01, 95% CI 1.98–2.03; adjusted for sociodemographic features 1.83, 95% CI 1.80–1.86). (Depressive and anxious symptoms demonstrated excellent reliability with Cronbach’s alpha of 0.92 (95% CI 0.92–0.92) for PHQ-9 and 0.88 (95% CI 0.88–0.88) for GAD-2).

Extending these analyses of total scores, we then sought to better understand the relationship between irritability and individual depressive and anxious symptoms. Specifically, we examined the extent to which individual depressive and anxiety symptoms were correlated with individual irritability items (Fig. S1a). In general, correlations were similar between items, with r values ranging from 0.38 to 0.64. We then examined the network structure of irritability, depressive, and anxious symptoms, as a means of visualizing their relationship beyond bivariate correlation, with each circle representing a single symptom and its size reflecting number of other symptoms with which it is correlated with r of at least 0.2. Network structure was somewhat different between individuals with and without moderate or greater major depressive disorder (*p*-value by permutation <0.001; Fig. S1b, S1c)—that is, the relationship between irritability and depressive or anxious symptoms differed depending on whether they occurred as part of a probable major depressive episode. In particular, the figures illustrate a stronger correlation between irritability and thoughts of suicide in the presence of at least moderate depression, but a weaker correlation with concentration and interest.

**Fig. 2:**
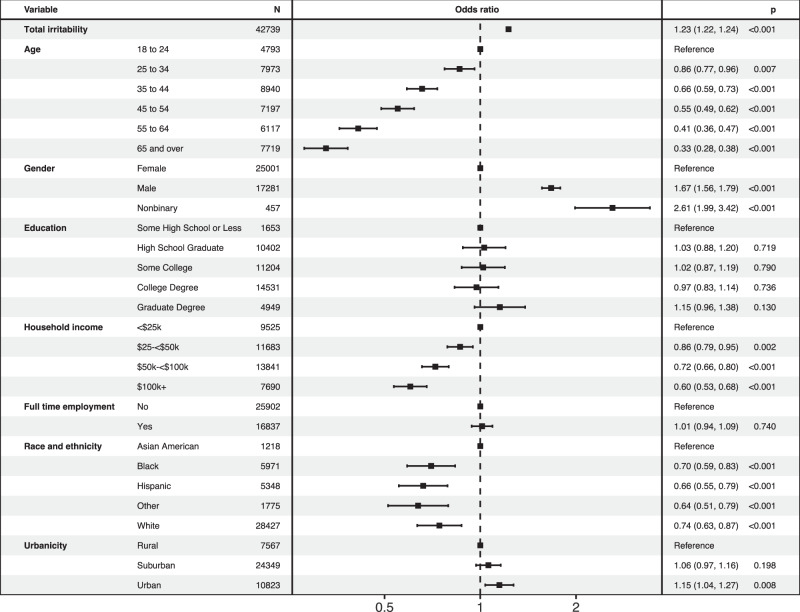
Logistic regression model of suicidality.

**Fig. 3:**
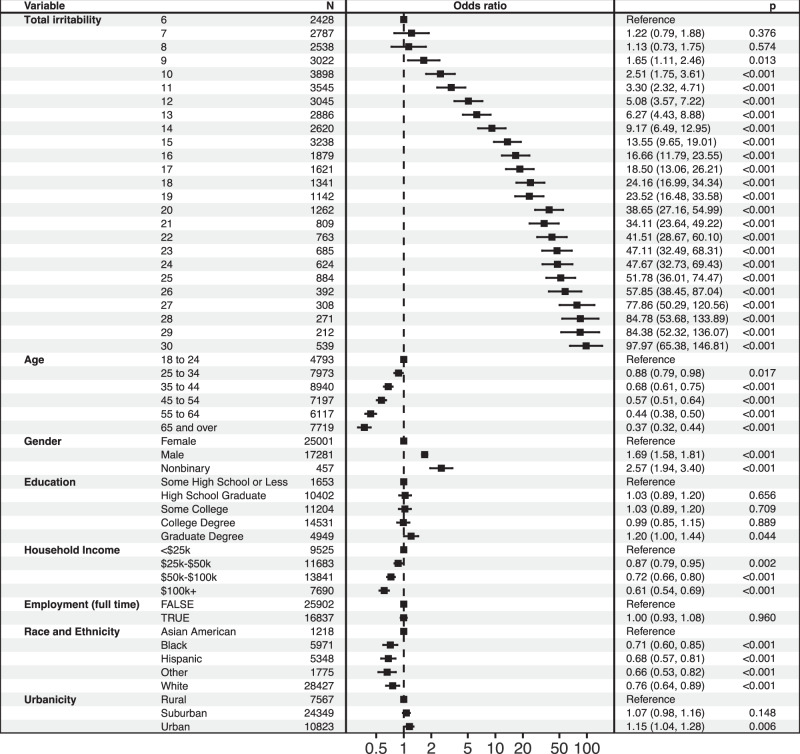
Logistic regression model of thoughts of suicide, examining irritability as categorical rather than linear variable. Note: Multivariable survey-weighted linear regression simultaneously incorporating all of the features included in the figure.

Among depressive symptoms, the relationship between irritability and thoughts of suicide has been demonstrated in clinical samples, so we next estimated the extent of association between irritability and suicidal thoughts in this population-based sample. Greater irritability was associated with likelihood of thoughts of suicide in univariate analysis (OR 1.24, 95% CI 1.24–1.25) as well as regression models adjusted for sociodemographic features (OR 1.23, 95% CI 1.22–1.24); Fig. 2. In follow-up analyses, we first sought to determine whether effects differed by age or gender strata. Adding interaction terms with either age or gender significantly improved model fit; effects of irritability were greatest among males (OR 1.25, 95% CI 1.24–1.26) and among those aged 65 and older (OR 1.37, 95% CI 1.33–1.41); Table S2. We then evaluated the possibility that the association between irritability and thoughts of suicide was non-linear, by considering irritability score as a categorical rather than linear variable: Fig. 3 illustrates this monotonic but greater-than-linear effect. Finally, we incorporated severity of depression and anxiety in the original sociodemographic-adjusted regression models, to examine the extent to which associations with irritability reflected more severe depressive or anxious symptoms. Associations persisted but magnitude was attenuated (OR 1.07, 95% CI 1.06–1.08).

In light of this cross-sectional association, we next sought to understand whether irritability preceded the emergence of suicidal ideation by examining a subset of survey participants who returned for a subsequent survey, yielding a longitudinal cohort. That is, among the 1979/2295 (86.2%) individuals in the initial survey wave who had not expressed thoughts of suicide and were assessed on the subsequent one (mean follow-up was 47.2 days [SD 8.6 days]), we examined the association between irritability at the first survey and emergence of thoughts of suicide at the next one. In logistic regression models, greater irritability on initial survey was significantly associated with greater likelihood of thoughts of suicide at follow-up (unadjusted OR 1.21, 95% CI 1.16–1.25, adjusted OR 1.17, 95% CI 1.12–1.23; Figure S2).

Finally, as there is no scale-defined threshold at which individuals are considered to have clinically significant irritability, we instead characterized those individuals who have levels of irritability in the top decile in the general population without at least moderate major depressive or anxious symptoms. In the weighted survey, 1.8% of these respondents had irritability in the top decile. Figure S3 illustrates sociodemographic features associated with this degree of irritability, which paralleled those for irritability in the sample as a whole: features associated with high irritability included younger age, female gender, lower education, and lower income.

## Discussion

In this survey of 42,739 adults in all 50 U.S. states, irritability was common, frequently but not always co-occurred with depressive and anxious symptoms, and was associated with greater likelihood of thoughts of suicide both at initial and follow-up survey completion.

In children and adolescents, irritability is recognized as a core feature of major depressive episodes [8, 12, 34], albeit a nonspecific one [35]. In this cohort, we find that irritability is greatest among young adults, and diminishes substantially with age. A key question for future work will be characterizing long-term changes in irritability, particularly among young adults with high levels of irritability, particularly in light of prior work indicating it is predictive of both depressive and anxiety disorders [14].

Beyond depressive symptoms, two prior reports from clinical trials of outpatients with major depressive disorder found an association between irritability and greater levels of anxiety [3, 6], similar to what we observed in the present study. On the other hand, a substantial subset of our cohort—representing ~1.8% of those without probable major depression or generalized anxiety—reported high levels of irritability. Sociodemographic features associated with high levels of irritability in the absence of depression or anxiety were similar to those that predicted greater irritability among the cohort as a whole—most notably younger age, female gender, lower education, and lower income. In line with the RDoC framework [15, 16], this result highlights the importance of examining how neuropsychiatric features vary across generalizable populations, not solely individual diagnoses, and how they vary from mild to severe. While negative valence symptoms are strongly correlated, substantial irritability can still occur in the absence of depression or anxiety—and our analysis of symptom networks suggests that the relationship with irritability may differ when depression and anxiety are less prominent. In particular, network analysis illustrates that the correlation of irritability with suicidal thoughts is stronger in the presence of moderate or greater depression, while the correlation with concentration and interest is less strong.

Prior work has also investigated the association between irritability and adverse outcomes in major depression [6, 8, 9], including suicidality [3, 10]. In reviewing this literature, Orri and colleagues note marked heterogeneity in such studies, compounded by generally weak assessments of irritability and cross-sectional designs [36]. The present study, drawing on both cross-sectional and longitudinal data and including a validated measure of irritability, supports this association in a representative population-based sample, further highlighting its public health significance. In particular, while irritability is most common among younger individuals, the magnitude of association between irritability and thoughts of suicide is, if anything, stronger among older individuals, underscoring the importance of broadening the focus on irritability beyond adolescents to adults. The prevalence of this symptom, and its association with suicidal thoughts, indicate the importance of incorporating measurement of irritability in clinical assessment, as well as the need to further investigate the extent to which treatments may address and ameliorate irritability.

### Limitations

Our study has multiple limitations. The cross-sectional components of the study preclude causal inference, and even longitudinal results must be interpreted with caution. Further, the web-based nonprobability design does not allow calculation of response rate and investigation of biases attributable to non-response, since individuals can opt-in to the survey after passively viewing multiple survey options provided by the vendor. However, we note that prior validation studies from this survey supported concordance both with probability-sampled polls and administrative data [37, 38]. Moreover, Pew Research data for 2023 [39] indicate that 88% of adults 65 and older use the internet regularly, along with 87% of those with income less than $30,000 per year, and 90% of those with a high school education or less. For individuals with psychiatric illness, including serious mental illness, one study similarly found that 88% reported internet use [40]. A strength of our study is the ability to oversample more challenging populations to ensure adequate coverage even if they have differential internet use.

As this study was not designed to investigate psychopathology more broadly, we did not assess current substance use, nor other psychiatric disorders commonly associated with irritability, including bipolar disorder. An important direction for further investigation will be characterizing the contribution of such disorders.

Finally, these surveys were conducted during a period when levels of depressive symptoms exceeded those observed prior to the COVID-19 pandemic. While such symptoms have generally been consistent in this and other surveys since 2020, the extent to which they reflect a particular set of stressors in a single country remains to be determined.

Conversely, we note multiple strengths, including a 50-state survey sample designed to be reflective of the US adult population, and the use of a validated measure of irritability optimal for adult rather than child and adolescent populations [21]. The large sample allows us to characterize the continuum of irritability in the general population, and to systematically control for sociodemographic features which could otherwise confound associations with outcomes such as suicidal thoughts.

## Conclusion

Taken together, our demonstrate the substantial prevalence of irritability among U.S. adults, confirming its association with both depressive and anxious symptoms, as well as thoughts of suicide in particular. In this context, the continued omission of irritability as a symptom of mood and anxiety disorders, and the lack of investigation of treatment for these symptoms, merits reconsideration.

## Supplementary information

Supplemental Materials and Methods

## Data Availability

The survey used for this study is available from the corresponding author for non-commercial use.
