# Supplementary material for: Prevalence and correlates of irritability among U.S. adults
Source: Neuropsychopharmacology. 2024 Aug 24;49(13):2052–9. doi: 10.1038/s41386-024-01959-3 (PMC11480215; doi:10.1038/s41386-024-01959-3)
Supplement: Supplementary file 1 — Supplemental Materials and Methods [file 41386_2024_1959_MOESM1_ESM.docx]

**Supplemental Materials and Methods**

Table of Contents

[Table S1. Unadjusted (univariate) associations between sociodemographic and clinical features and irritability score. 2](#_Toc171944431)

[Figure S1a. Correlations between individual depressive, anxious, and irritable symptoms as a heat map reflecting strength of correlations 3](#_Toc171944432)

[Figure S1b. Network model: Depression and anxiety symptoms for individuals with PHQ9 10 or greater 4](#_Toc171944433)

[Figure S1c. Network model: Depression and anxiety symptoms for individuals with PHQ9 less than 10 5](#_Toc171944434)

[Table S2. Survey-weighted logistic regression models of association between suicidal ideation and irritability, adjusted for sociodemographic features, stratified by age or gender 6](#_Toc171944435)

[Figure S2. Logistic regression model of thoughts of suicide at subsequent survey 7](#_Toc171944436)

[Figure S3. Logistic regression model of high irritability 8](#_Toc171944437)

# **Table S1.** Unadjusted (univariate) associations between sociodemographic and clinical features and irritability score.

| **Feature** | **Category** | **Estimate*** | **Lower CI** | **Upper CI** |
| --- | --- | --- | --- | --- |
| Age | Age 18 to 24 | Reference |  |  |
|  | Age 25 to 34 | -0.36 | -0.60 | -0.12 |
|  | Age 35 to 44 | -1.28 | -1.52 | -1.05 |
|  | Age 45 to 54 | -1.82 | -2.07 | -1.57 |
|  | Age 55 to 64 | -3.63 | -3.87 | -3.39 |
|  | Age 65 and older | -5.54 | -5.76 | -5.32 |
| Gender | Female | Reference |  |  |
|  | Male | -0.65 | -0.78 | -0.53 |
|  | Nonbinary | 2.44 | 1.85 | 3.04 |
| Race and ethnicity | Asian | Reference |  |  |
|  | Black | 0.26 | -0.10 | 0.61 |
|  | Hispanic | 0.66 | 0.30 | 1.02 |
|  | Other Race or Ethnicity | 0.68 | 0.23 | 1.12 |
|  | White | -0.06 | -0.39 | 0.26 |
| Education | Less than H.S. | Reference |  |  |
|  | High school | -1.77 | -2.11 | -1.43 |
|  | Some college | -1.87 | -2.20 | -1.53 |
|  | College degree | -2.74 | -3.07 | -2.41 |
|  | Graduate degree | -3.37 | -3.72 | -3.02 |
| Employment | Full time | 0.63 | 0.51 | 0.75 |
| Household Income | < $25k | Reference |  |  |
|  | $25k - <$50k | -1.22 | -1.41 | -1.04 |
|  | $50k - <$100k | -1.72 | -1.89 | -1.55 |
|  | $100k+ | -2.20 | -2.39 | -2.01 |
| Urbanicity | Rural | Reference |  |  |
|  | Suburban | -0.55 | -0.72 | -0.38 |
|  | Urban | -0.49 | -0.68 | -0.30 |
| PHQ9 | Total score | 0.59 | 0.59 | 0.60 |
| GAD2 | Total score | 2.01 | 1.98 | 2.03 |
|  |  |  |  |  |
| PHQ9, Patient Health Questionnaire, 9-item;  GAD2, Generalized Anxiety Disorder, 2-item | | | | |

# **Figure S1a.** Correlations between individual depressive, anxious, and irritable symptoms as a heat map reflecting strength of correlations

# **Figure S1b.** Network model: Depression and anxiety symptoms for individuals with PHQ9 10 or greater

Node sizes indicate number of edges (connections) to other nodes (symptoms). Edge weight indicates strength of correlation between nodes. Figure indicates correlations of 0.2 or greater.

# **Figure S1c.** Network model: Depression and anxiety symptoms for individuals with PHQ9 less than 10

Node sizes indicate number of edges (connections) to other nodes (symptoms). Edge weight indicates strength of correlation between nodes. Figure indicates correlations of 0.2 or greater.

# **Table S2.** Survey-weighted logistic regression models of association between suicidal ideation and irritability, adjusted for sociodemographic features, stratified by age or gender

|  | **OR** | **Lower CI** | **Upper CI** |  |  |  |
| --- | --- | --- | --- | --- | --- | --- |
| **Gender** |  |  |  | 2logLR = 19.75, p<.0001 | |  |
| *Male* | 1.25 | 1.24 | 1.26 |  |  |  |
| *Female* | 1.21 | 1.20 | 1.22 |  |  |  |
| *Nonbinary* | 1.26 | 1.19 | 1.32 |  |  |  |
| **Age** |  |  |  | 2logLR = 125.83, p<.0001 | |  |
| *18 to 24* | 1.18 | 1.16 | 1.20 |  |  |  |
| *25 to 34* | 1.21 | 1.20 | 1.22 |  |  |  |
| *35 to 44* | 1.23 | 1.21 | 1.24 |  |  |  |
| *45 to 54* | 1.25 | 1.23 | 1.27 |  |  |  |
| *55 to 64* | 1.27 | 1.25 | 1.30 |  |  |  |
| *65 and over* | 1.37 | 1.33 | 1.41 |  |  |  |
|  |  |  |  |  |  |  |
| Survey-weighted logistic regression models adjusted for age, gender, employment status, household income, race and ethnicity, education, and urbanicity | | | | | | |
| OR, odds ratio; CI, 95% confidence interval | | | |  |  |  |

# **Figure S2.** Logistic regression model of thoughts of suicide at subsequent survey

* Excludes n=7 individuals who identify as nonbinary, for whom OR ~0.

# **Figure S3.** Logistic regression model of high irritability
